# Proteomics reveals a core molecular response of *Pseudomonas putida *F1 to acute chromate challenge

**DOI:** 10.1186/1471-2164-11-311

**Published:** 2010-05-19

**Authors:** Dorothea K Thompson, Karuna Chourey, Gene S Wickham, Stephanie B Thieman, Nathan C VerBerkmoes, Bing Zhang, Andrea T McCarthy, Matt A Rudisill, Manesh Shah, Robert L Hettich

**Affiliations:** 1 Department of Biological Sciences, Purdue University, 915 W. State Street, West Lafayette, IN 47907, USA; 2 Chemical Sciences Division, Oak Ridge National Laboratory, Oak Ridge, TN 37831, USA; 3 Department of Biomedical Informatics, Vanderbilt University, Nashville, TN 37232, USA; 4 Biosciences Division, Oak Ridge National Laboratory, Oak Ridge, TN 37831, USA

## Abstract

**Background:**

*Pseudomonas putida *is a model organism for bioremediation because of its remarkable metabolic versatility, extensive biodegradative functions, and ubiquity in contaminated soil environments. To further the understanding of molecular pathways responding to the heavy metal chromium(VI) [Cr(VI)], the proteome of aerobically grown, Cr(VI)-stressed *P*. *putida *strain F1 was characterized within the context of two disparate nutritional environments: rich (LB) media and minimal (M9L) media containing lactate as the sole carbon source.

**Results:**

Growth studies demonstrated that F1 sensitivity to Cr(VI) was impacted substantially by nutrient conditions, with a carbon-source-dependent hierarchy (lactate > glucose >> acetate) observed in minimal media. Two-dimensional HPLC-MS/MS was employed to identify differential proteome profiles generated in response to 1 mM chromate under LB and M9L growth conditions. The immediate response to Cr(VI) in LB-grown cells was up-regulation of proteins involved in inorganic ion transport, secondary metabolite biosynthesis and catabolism, and amino acid metabolism. By contrast, the chromate-responsive proteome derived under defined minimal growth conditions was characterized predominantly by up-regulated proteins related to cell envelope biogenesis, inorganic ion transport, and motility. TonB-dependent siderophore receptors involved in ferric iron acquisition and amino acid adenylation domains characterized up-regulated systems under LB-Cr(VI) conditions, while DNA repair proteins and systems scavenging sulfur from alternative sources (e.g., aliphatic sulfonates) tended to predominate the up-regulated proteome profile obtained under M9L-Cr(VI) conditions.

**Conclusions:**

Comparative analysis indicated that the core molecular response to chromate, irrespective of the nutritional conditions tested, comprised seven up-regulated proteins belonging to six different functional categories including transcription, inorganic ion transport/metabolism, and amino acid transport/metabolism. These proteins might potentially serve as indicators of chromate stress in natural microbial communities.

## Background

*Pseudomonas putida *is a ubiquitous gram-negative, saprophytic bacterium belonging to the gamma class of the *Proteobacteria*. Endowed with a remarkable environmental adaptability, *P*. *putida *strain F1 [1], for example, has been investigated most extensively as a model organism for the microbial degradation of such xenobiotic aromatic compounds as toluene, benzene, and ethylbenzene [2]. Considerably less scientific focus has been devoted to elucidating the molecular basis of heavy metal resistance functions exhibited by *P*. *putida *strains. Genomic analysis of *P*. *putida *strain KT2440 revealed an unexpectedly large repertoire of genes predicted to be involved in metal homeostasis, tolerance, and resistance, inferring that the organism is habituated to heavy metal exposure in its environment [3]. *P*. *putida *strains, for instance, have been isolated from the effluent of previously operated mercury-reducing bioreactors [4], and *Pseudomonas *spp. were reported to be dominant members of heavy metal-contaminated U.S. Department of Energy (DOE) sites, including a subsurface paleosol microbial community [5] and uranium-contaminated groundwater environment [6].

Hexavalent chromium [Cr(VI)], in the form of chromate (CrO_4_^2-^) or dichromate (Cr_2_O_7_^2-^), is a widely distributed environmental contaminant due to its prevalent use in industrial and military defense applications [7,8]. Microbially mediated reduction of soluble, toxic Cr(VI) to sparingly soluble, less toxic Cr(III) is a potentially promising strategy for the *in situ *remediation of Cr(VI)-contaminated subsurface environments. Priester *et al. *[9] demonstrated that unsaturated biofilms of *P. putida *strain mt-2 on membranes overlaying iron-deficient solid media either containing dichromate or solid dichromate-coated hematite were able to completely reduce Cr(VI) under both conditions. Additionally, efficient and rapid reduction of Cr(VI) to Cr(III) has been measured for a soluble flavoprotein (ChrR) purified and kinetically characterized from *P*. *putida *MK1 [10,11] and for soluble crude fractions prepared from *P*. *putida *PRS2000 [12], suggesting that *P*. *putida*, if present in the indigenous microbial community, is likely an important contributor to the bioreduction of chromate in polluted soil environments. Surprisingly, there is a paucity of information on the physiological and global molecular response of *P*. *putida *to exogenous metals such as chromate, despite the fact that this soil bacterium has been used as a model organism for vadose zone bioremediation in various studies [9,13,14]. The availability of the genome sequences for *P*. *putida *strains F1 [DOE Joint Genome Institute (JGI); http://genome.jgi-psf.org/finished_microbes/psepu/psepu.home.html] and KT2440 [15] provides an opportunity to access its metabolic versatility and explore the molecular basis for its enhanced ability to adapt to the various environmental conditions present in pristine and metal-contaminated soils.

Here we define the growth sensitivity of *P*. *putida *F1 to chromate when cultivated in complex (Luria Bertani) medium compared to defined minimal medium containing three different single carbon and energy sources (glucose, lactate, or acetate). Using a liquid-chromatography mass spectrometry-based "bottom-up" proteomic approach, we analyzed the global proteome profiles of F1 in an attempt to provide insight into the molecular systems underlying the cellular stress response to an acute exposure of chromate. In general, the chromate-perturbed F1 proteome was markedly different depending on whether challenged cells were cultivated in LB broth or minimal medium with lactate as the single carbon substrate. A core protein response to chromate, irrespective of the nutritional environment tested, is described and may provide potential *in situ *biomarkers for environmental biomonitoring of heavy metal stress in natural microbial communities.

## Results

### Impact of nutritional environment on *P. putida *growth and Cr(VI) reduction

**Figure 1:**
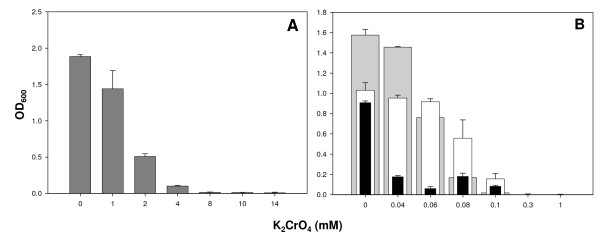
**Effect of carbon source on MIC of chromate for *P. putida *F1**. Optical densities (OD_600_) of F1 cultures after 48 h of growth in the presence of varying concentrations of chromate (K_2_CrO_4_) are shown. (A) F1 growth in LB broth. (B) F1 growth in M9 minimal media supplemented with 50 mM glucose (grey bars), 50 mM sodium lactate (white bars), or 67 mM sodium acetate (black bars) as the sole source of carbon. All cultures were grown in triplicate. Error bars denote the standard deviation of replicate measurements.

To assess the physiological impact of nutrient conditions on *P*. *putida *F1 sensitivity to Cr(VI), we determined the minimal inhibitory concentrations (MICs) of chromate for F1 in LB and M9 minimal media supplemented with 50 mM glucose (M9G), 50 mM sodium lactate (M9L), or 67 mM sodium acetate (M9A) as the sole carbon source in the presence of K_2_CrO_4 _at concentrations ranging from 0.04 to 14 mM. F1 cells exhibited markedly different tolerance responses to chromate depending on the nutritional environment. The MIC of Cr(VI) for LB-propagated F1 cells was >4 mM (Figure 1A), an approximately 40-fold greater concentration than that observed for F1 cells grown in M9 media irrespective of carbon source (MIC > 0.1 mM) (Figure 1B). Among the different minimal media, the nature of the carbon source influenced the growth ability of F1 in the presence of chromate. Higher ODs were measured for M9G in the absence of and at the lowest Cr(VI) concentration (0.04 mM) tested than for M9L and M9A. As Cr(VI) concentrations were increased, however, M9L supported growth to higher cell densities than either M9G or M9A, and at the highest levels of chromate tested, 0.08 mM and 0.1 mM, cell densities in M9L surpassed those in M9G by 3- and 9-fold, respectively (Figure 1B). Thus, there was a carbon-source-dependent hierarchy in chromate-amended M9 media (lactate > glucose >> acetate) that cannot be explained simply on the basis of better growth medium energetics since glucose supported higher cell densities than lactate in the absence of Cr(VI), yet as Cr(VI) levels increased lactate supported higher cell densities than glucose. Therefore, M9L was chosen as the minimal medium for subsequent growth studies.

**Figure 2:**
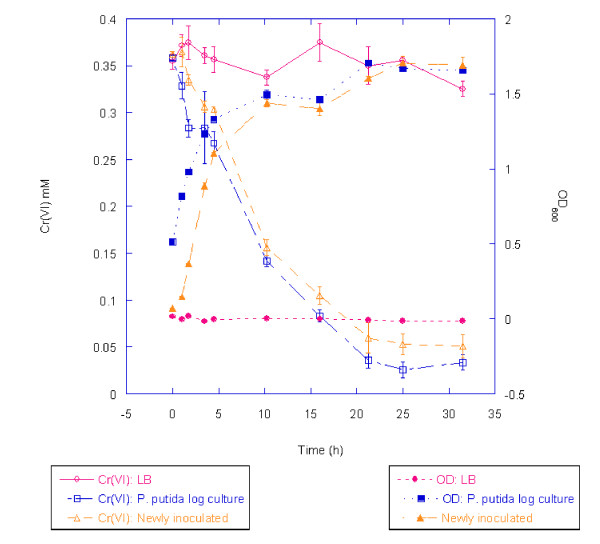
**Chromate reduction by *P. putida *F1**. The progression of reduction of 0.3 mM K_2_CrO_4 _by *P*. *putida *F1 in LB broth is shown over a 31.5 h period. At T = 0, F1 cells were either freshly inoculated into the chromate-containing growth medium from an overnight culture to an OD of 0.07 (open triangles) or chromate was added to a culture of F1 already in mid-log phase (open squares). The abiotic control is also shown (red open circles). Each data point represents the mean of three replicate measurements. Error bars indicate one standard deviation.

To determine whether F1 is capable of chromate reduction, cells were grown in LB broth containing K_2_CrO_4 _and chromate reduction was measured for up to 31.5 h. F1 reduced 0.3 mM Cr(VI) to 0.05 mM or less within 25 h (Figure 2). The ability of F1 to carry out this reduction was largely independent of the initial cell density of the culture as evidenced by the fact that both a culture grown to mid-log phase (OD = 0.5) prior to chromate exposure and a newly inoculated culture (OD = 0.07) both reduced chromate to similar final concentrations (Figure 2). In addition, we tested reduction ability at 1 mM chromate by mid-log cells and found that there was no reduction within the first 75 min (initial chromate concentration and concentration at 75 min measured at 0.93 ± 0.01 and 0.92 ± 0.04 mM, respectively); furthermore, at this higher initial concentration there was less reduction over a 24 h period (final concentration = 0.77 mM ± 0.03 mM).

**Figure 3:**
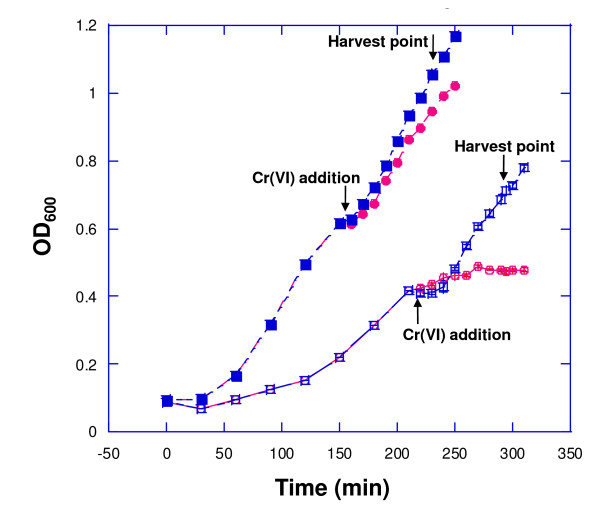
**Impact of chromate challenge on *P. putida *F1 growth in LB versus M9L media**. The optical densities (OD_600_) of F1 cultures grown aerobically under LB (closed symbols) versus defined minimal M9L media (open symbols) in either the absence or presence of 1 mM K_2_CrO_4 _are shown: LB-grown cells with no chromate (closed squares), LB-grown cells with chromate added to 1 mM at the mid-log point (closed circles), M9L-grown cells with no chromate (open squares), and M9L-grown cells with chromate added to 1 mM at the mid-log point (open circles). The point of Cr(VI) addition and cell harvesting for proteomic analyses is presented. Each data point represents the mean of three replicate samples. Error bars indicate one standard deviation.

A chromate level of 1 mM was selected for challenging mid-log-phase LB- and M9L-grown cells, because it represented the lowest concentration tested for which cell toxicity was observed under LB conditions. Because we were interested in delineating the early molecular response to Cr(VI) exposure, the 75-min time interval following chromate exposure was selected for harvest and analysis of F1 proteomes. Figure 3 shows the growth differences between the Cr(VI)-treated and untreated cultures for the two different nutrient conditions at the collection time interval (75 min post-chromate addition) for shotgun proteomics analysis. The effect of Cr(VI) addition was greater on M9L cells, which showed a ~34% reduction in cell density at the harvest point, while the growth difference between Cr(VI)-treated and untreated control cells in LB liquid media was ~10%. No measurable Cr(VI) reduction by F1 was detected under either growth condition within the 75-min period of exposure, although this strain of *P*. *putida *was shown to reduce 0.3 mM chromate to ~0 mM over 24 h (Figure 2).

### Global analysis of *P. putida *F1 proteomes under different growth conditions

Protein abundance measurements were compared for Cr(VI)-challenged and untreated *P*. *putida *F1 cells grown aerobically in either complex (LB) medium or defined minimal (M9L) medium containing lactate as the sole carbon/energy source in order to assess growth condition-dependent differences in the molecular response to acute chromate exposure as well as proteomic signatures indicative of heavy metal stress irrespective of the nutritional milieu. In general, a total of 2140 (41%) and 2383 (45%) functionally diverse, non-redundant proteins out of 5252 predicted proteins were detected in *P*. *putida *F1 cultivated in LB or M9L media, respectively, with and without chromate. By pooling all of the non-redundant proteins across both minimal and LB media samples, a total of 2,681 proteins were detected, which is ~50% of the predicted proteome just from these four growth conditions. The entire list of identified proteins with their spectral counts and associated statistical analyses can be found in Additional file 1. A total of 946 proteins were found in all growth states and technical replicates (Additional file 2). Further analysis indicated that 257 proteins were exclusively expressed in LB-grown cell samples, while 485 proteins were unique to M9L-grown cells (Additional file 3).

**Figure 4:**
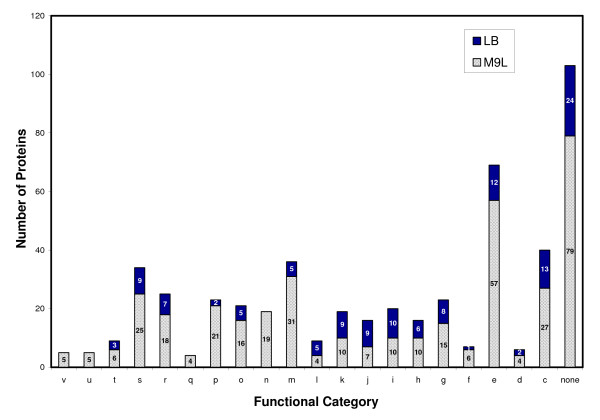
**Functional category distribution of proteins identified from F1 cells grown in LB or M9L media in the absence of chromate**. Letters along the x-axis refer to the following functional role categories: (v) defense mechanisms; (u) intracellular trafficking, secretion and vesicular transport; (t) signal transduction mechanisms; (s) unknown function; (r) general function prediction; (q) secondary metabolite biosynthesis, transport and catabolism; (p) inorganic ion transport and metabolism; (o) post-translational modification, protein turnover, and chaperones; (n) cell motility; (m) cell wall membrane and envelope biogenesis; (l) DNA replication, recombination and repair; (k) transcription; (j) translation of ribosomal structure and biogenesis; (i) lipid transport and metabolism; (h) coenzyme transport and metabolism; (g) carbohydrate transport and metabolism; (f) nucleotide transport and metabolism; (e) amino acid transport and metabolism; (d) cell cycle control, cell division and chromosome partitioning; (c) energy production and conversion; and (none) no specific function. The number of proteins identified for each COG category under the different growth conditions is presented within the split vertical bars.

Statistically significant proteins, *i.e.*, those exhibiting at least a two-fold change in expression and an FDR < 0.01, in LB- and M9L-cultivated control cells had a broad functional distribution (Figure 4 and Additional file 4). Not unexpectedly, control cells propagated in M9L medium, which is much less nutritionally robust than LB, showed a much higher proportion of protein expression in the COG categories of cell wall membrane and envelope biogenesis (m), amino acid transport and metabolism (e), and energy production and conversion (c) (Figure 4). Enzymes and transport proteins, specifically D-lactate dehydrogenase (cytochrome; Pput 4603) and L-lactate transporter (permease; Pput 4601), related to lactate utilization were only identified in cell samples grown with this carbon source (Additional file 1), which was a mixture of the D and L stereoisomers. Lactate is converted to pyruvate by lactate dehydrogenase and then is further metabolized via the tricarboxylic acid (TCA) cycle. In addition, pyruvate dehydrogenase (Pput 0594), involved in catalyzing the oxidative decarboxylation of pyruvate to acetyl-CoA and CO_2_, and two other enzymes of the TCA cycle, NADP-dependent isocitrate dehydrogenase (Pput 1822) and malate dehydrogenase (Pput 4958), had substantially decreased abundance levels under chromate conditions in M9L medium. The reduced synthesis of these key primary metabolic enzymes is consistent with the more severe Cr(VI)-induced growth defects observed for F1 cells in M9L media (Figure 3).

Hypothetical proteins (s) and poorly characterized proteins [*i.e.*, those with general functions (r) or no specific function (none)] constituted the largest proportion of the two control proteome datasets, with 31% for the LB samples and 32% for the M9L samples (Figure 4). For those genes showing no similarity to sequences with known functions, this study provides evidence of their actual expression and suggests clues to their possible biological roles in F1. Expression of proteins involved in cell motility (n) characterized the global protein profile of M9L-grown control cells but were not represented in the LB sub-proteome. Similarly, proteins belonging to the following COG categories were not identified in LB control samples: defense mechanisms (v); intracellular trafficking, secretion and vesicular transport (u); and secondary metabolite biosynthesis, transport and catabolism (q) (Figure 4).

### Differential expression in chromate-stressed LB-derived proteome

Comparative analysis of the Cr(VI)-challenged and control LB samples revealed a total of 66 proteins exhibiting a significant reproducible change in abundance in response to Cr(VI) treatment, with 44 of those proteins up-regulated and 22 down-regulated (Table 1). Prominent up-regulated proteins included three periplasmic binding proteins (Pput 1968, Pput 4553, Pput 0948), methyltransferase type 11 (Pput 0942), fumarate lyase (FumC, Pput 0984), the antioxidative enzyme superoxide dismutase (Fe-Mn family, Pput 0985), haem oxygenase (Pput 1042), a TonB-dependent hemoglobin/transferrin/lactoferrin family receptor (Pput 1043), a 2-oxoglutarate (2OG)- and Fe(II)-dependent oxygenase (Pput 0892), and a functionally unknown two-component transcriptional regulator of the winged helix family (Pput 0287) (Table 1). Additionally, five amino acid adenylation domain-containing proteins (Pput 1624, Pput 1674-1676, and 1680) encoded within a gene cluster showed increased synthesis in response to chromate stress only under LB growth conditions (not M9L). In general, the most frequently represented COG functional group in the up-regulated LB-derived proteome dataset was inorganic ion transport and metabolism (12 proteins out of 44). Proteins belonging to this role category were associated predominantly with iron homeostasis or metabolism (*e.g*., TonB-dependent siderophore receptors Pput 0891, 1043, 1678, 1714; haem oxygenase Pput 1042; and TonB system transport protein ExbD) (Table 1).

**Table 1:** Differentially expressed proteins identified in Cr(VI)-challenged LB-grown F1

Gene ID	Functional category	Protein function	Poisson regression coefficient	FDR
Pput 4553	Inorganic ion transport and metabolism	periplasmic binding protein	26.59	3.89E-17
Pput 0942	Coenzyme transport and metabolism	Methyltransferase type 11	26.54	2.60E-16
Pput 1043	Inorganic ion transport and metabolism	TonB-dependent hemoglobin/transferrin/lactoferrin family receptor	25.63	1.59E-06
Pput 4550	Unknown Function	protein of unknown function DUF399	25.29	9.88E-05
Pput 0984	Energy production and conversion	fumarate lyase	25.06	3.01E-27
Pput 1680	Secondary metabolites biosynthesis, transport and catabolism	amino acid adenylation domain	24.97	1.82E-03
Pput 1675	Secondary metabolites biosynthesis, transport and catabolism	amino acid adenylation domain	24.84	6.16E-08
Pput 1968	Inorganic ion transport and metabolism	periplasmic solute binding protein	24.76	2.34E-07
Pput 1678	Inorganic ion transport and metabolism	TonB-dependent siderophore receptor	24.38	4.17E-05
Pput 1640	Amino acid transport and metabolism	2,4-diaminobutyrate 4-transaminase	23.81	9.88E-08
Pput 1961	Unknown Function	MbtH domain protein	23.60	2.47E-06
Pput 5216	Intracellular trafficking, secretion and vesicular transport	TonB system transport protein ExbD type-1	23.42	2.62E-05
Pput 0892	Unknown Function	2OG-Fe(II) oxygenase	23.24	1.54E-04
Pput 5104	Energy production and conversion	Ubiquinone biosynthesis hydroxylase, UbiH/UbiF/VisC/COQ6 family	22.97	1.82E-03
Pput 3040	Cell Motility	hypothetical protein	22.78	1.55E-07
Pput 0943	none	lipopolysaccharide kinase	22.76	7.08E-03
Pput 0603	Unknown Function	hypothetical protein	22.76	7.08E-03
Pput 1679	Unknown Function	protein of unknown function DUF323	22.76	7.08E-03
Pput 3610	Cell wall membrane and envelope biogenesis	Lytic transglycosylase, catalytic	22.76	7.08E-03
Pput 1973	Secondary metabolites biosynthesis, transport and catabolism	Lysine/ornithine N-monooxygenase-like protein	22.76	2.34E-07
Pput 0636	Coenzyme transport and metabolism	aminotransferase class-III	-0.73	9.20E-03
Pput 3436	Carbohydrate transport and metabolism	methylisocitrate lyase	-0.74	6.72E-03
Pput 0936	Energy production and conversion	hydro-lyase, Fe-S type, tartrate/fumarate subfamily, alpha subunit	-0.76	2.05E-12
Pput 2460	Post translational modification and protein turnover chaperones	heat shock protein Hsp20	-0.80	1.67E-03
Pput 1617	Energy production and conversion	cytochrome c oxidase, cbb3-type, subunit II	-0.90	1.30E-05
Pput 1610	Energy production and conversion	cytochrome c oxidase, cbb3-type, subunit III	-0.92	4.85E-05
Pput 1615	Energy production and conversion	cytochrome c oxidase, cbb3-type, subunit III	-0.92	2.47E-06
Pput 1123	Inorganic ion transport and metabolism	bacterioferritin	-1.06	9.58E-03
Pput 2593	Lipid transport and metabolism	3-oxoacid CoA-transferase, B subunit	-1.16	3.81E-03
Pput 4498	Lipid transport and metabolism	acetyl-CoA acetyltransferase	-1.23	3.34E-07
Pput 4329	Amino acid transport and metabolism	thiamine pyrophosphate enzyme TPP binding domain protein	-1.25	4.49E-03
Pput 1388	Amino acid transport and metabolism	putative agmatinase	-1.82	8.75E-11
Pput 0143	Energy production and conversion	cytochrome c, class I	-1.84	5.95E-09
Pput 2061	Inorganic ion transport and metabolism	catalase/peroxidase HPI	-2.01	8.74E-07
Pput 3734	Energy production and conversion	NADH:flavin oxidoreductase/NADH oxidase	-2.60	1.37E-04
Pput 2594	Lipid transport and metabolism	3-oxoacid CoA-transferase, A subunit	-3.14	7.15E-09
Pput 2025	Transcription	transcriptional regulator, GntR family	-19.95	9.31E-03
Pput 1948	none	cytochrome c, class I	-22.49	1.37E-04
Pput 1559	Transcription	putative transcriptional regulator, AsnC family	-22.95	9.31E-03
Pput 2382	Energy production and conversion	Gluconate 2-dehydrogenase	-23.20	1.83E-03
Pput 3735	Transcription	AraC-type transcriptional regulator domain protein	-24.08	4.39E-03
Pput 1496	Signal transduction mechanisms	Two-component, sigma54 specific, transcriptional regulator, Fis family	-24.95	9.31E-03

FDR: False Discovery Rate

Table 1 lists top 20 up-regulated and down-regulated proteins in LB-grown chromate shocked cells of *P*. *putida *when compared to unstressed LB grown cells. Poisson regression coefficient score and False Discovery Rate (FDR) of 1% were used for analyzing expression levels of the proteins.

Down-regulated proteins constituted a small subset dominated by proteins with annotated functions in energy production and transcriptional regulation or signal transduction mechanisms (Table 1). Whereas superoxide dismutase (Pput 0985) was preferentially up-regulated under Cr(VI) challenge, the relative abundance of catalase/peroxidase HPI (Pput 2061), a scavenger of hydrogen peroxide (H_2_O_2_), decreased in Cr(VI)-exposed cells compared to the untreated control cells. Only one protein (Pput 1123) involved in iron metabolism or homeostasis was identified as being down-regulated in the LB proteome under chromate conditions in marked contrast to the numerous proteins of similar function shown to be up-regulated under identical conditions.

### Differential expression in chromate-stressed M9L-derived proteome

A total of 234 proteins exhibited statistically significant altered abundance (at least a 2-fold change and an FDR of 0.01) in response to acute chromate challenge under M9L growth conditions (Table 2 and Additional file 5). Among these, 95 were uniquely identified with dramatic changes in their expression levels under chromate challenge or control conditions (Table 2). Similar to the LB proteome dataset, the majority of differentially expressed proteins were identified as being up-regulated (130 proteins), while a smaller proportion (104 proteins) were down-regulated. In general, for the up-regulated sub-proteome, the most affected COG functional groups were those containing proteins of unknown function or no specific assigned function (3 proteins), as well as those with annotated functions in cell wall membrane and envelope biogenesis (17 proteins), inorganic ion transport/metabolism (14 proteins), and cell motility (16 proteins). In contrast to the differential protein profile generated under LB-chromate conditions, M9L proteins grouping in the role category of inorganic ion transport/metabolism were not specifically related to Fe uptake and utilization based on sequence annotation but instead had functions in the uptake of alternative sulfur sources or unknown substrates (e.g., ABC transporter related). Prominently up-regulated proteins involved in sulfur uptake and sulfur assimilatory metabolism included a taurine dioxygenase (TauD, Pput 0190), an ABC-type nitrate/sulfonate/bicarbonate transport system component (Pput 0191), and an aliphatic sulfonates family ABC transporter (Pput 2245) (Table 2 and Additional file 5). These proteins are involved in scavenging sulfur from alternative sulfur sources such as aliphatic sulfonates (*e.g*., taurine). Specifically, TauD catalyzes the oxygenolytic release of sulfite from taurine [16,17], thereby allowing sulfonates to enter the assimilatory sulfate reduction pathway at the stage of sulfite [18].

**Table 2:** Selected differentially expressed proteins detected in Cr(VI)-challenged M9L-grown F1

Gene ID	Functional category	Protein function	Poisson regression coefficient	FDR
Pput 0191	Inorganic ion transport and metabolism	ABC-type nitrate/sulfonate/bicarbonate transport systems periplasmic components-like protein	26.45	3.65E-14
Pput 0190	Secondary metabolites biosynthesis	Taurine dioxygenase	25.54	2.01E-15
Pput 4621	none	hypothetical protein	25.52	8.56E-06
Pput 1872	Cell Motility	methyl-accepting chemotaxis sensory transducer	24.87	2.68E-03
Pput 0391	Coenzyme transport and metabolism	biotin biosynthesis protein BioC	24.69	8.28E-07
Pput 3604	Unknown Function	protein of unknown function DUF1653	24.67	7.60E-03
Pput 1104	Transcription	transcriptional regulator, LysR family	24.67	7.60E-03
Pput 2545	Transcription	transcriptional regulator, LysR family	24.67	7.60E-03
Pput 2963	Replication	ATPase involved in DNA repair-like protein	24.67	7.60E-03
Pput 4712	Post translational modification and protein turnover chaperones	band 7 protein	24.67	7.60E-03
Pput 1882	Amino acid transport and metabolism	ABC transporter related	24.67	1.80E-04
Pput 1060	Carbohydrate transport and metabolism	glucose-6-phosphate 1-dehydrogenase	24.25	3.38E-10
Pput 0247	Inorganic ion transport and metabolism	ABC transporter related	24.11	4.50E-03
Pput 0999	Secondary metabolites biosynthesis	Mammalian cell entry related domain protein	23.78	4.50E-03
Pput 4172	Replication	Ribonuclease H	23.78	4.50E-03
Pput 3319	none	fatty acid cistrans isomerase	23.78	4.50E-03
Pput 0949	Energy production and conversion	protein of unknown function DUF1111	23.78	4.50E-03
Pput 4281	Coenzyme transport and metabolism	putative methyltransferase	23.78	4.50E-03
Pput 3991	Cell wall membrane and envelope biogenesis	MscS Mechanosensitive ion channel	23.78	4.50E-03
Pput 5174	Replication	ATP-dependent DNA helicase Rep	23.78	4.50E-03
Pput 2568	Amino acid transport and metabolism	FAD dependent oxidoreductase	-22.82	2.99E-07
Pput 1812	General function prediction	alpha/beta hydrolase fold	-23.83	2.79E-03
Pput 4237	Amino acid transport and metabolism	spermidine/putrescine ABC transporter ATPase subunit	-23.94	7.11E-29
Pput 2061	Inorganic ion transport and metabolism	catalase/peroxidase HPI	-24.23	2.03E-04
Pput 1736	Energy production and conversion	proton-translocating NADH-quinone oxidoreductase, chain L	-24.31	1.13E-12
Pput 0594	Energy production and conversion	Pyruvate dehydrogenase (acetyl-transferring)	-24.40	1.48E-14
Pput 3083	Energy production and conversion	aldehyde dehydrogenase	-24.55	5.31E-03
Pput 0110	General function prediction	Carbonic anhydrase/acetyltransferase isoleucine patch superfamily-like protein	-24.82	5.31E-03
Pput 4889	Amino acid transport and metabolism	phosphoribosyl-ATP pyrophosphohydrolase	-24.82	1.47E-03
Pput 0527	Amino acid transport and metabolism	L-seryl-tRNA(Sec) selenium transferase	-25.04	3.99E-04
Pput 4915	Carbohydrate transport and metabolism	glycogen/starch/alpha-glucan phosphorylase	-25.23	2.03E-04
Pput 1789	None	hypothetical protein	-25.31	5.33E-06
Pput 0932	General function prediction	intracellular protease, PfpI family	-25.63	2.52E-06
Pput 2569	None	hypothetical protein	-25.68	2.52E-06
Pput 4267	General function prediction	transferase hexapeptide repeat containing protein	-25.68	2.52E-06
Pput 1947	Energy production and conversion	cytochrome c, class I	-25.68	1.31E-06
Pput 2567	Amino acid transport and metabolism	extracellular solute-binding protein, family 1	-25.74	7.04E-08
Pput 2463	Cell wall membrane and envelope biogenesis	GCN5-related N-acetyltransferase	-25.92	3.86E-09
Pput 0592	Energy production and conversion	alpha/beta hydrolase fold	-26.07	1.94E-09
Pput 1187	None	hypothetical protein	-26.11	3.65E-14

FDR: False Discovery Rate

The table shows top 20 up-regulated and down-regulated proteins in Cr-shocked, M9L-grown cells of *P*. *putida *when compared to unstressed M9L cells. Proteome datasets were analyzed using the Poisson regression model combined with False Discovery Rate (FDR) of 1%.

Another conspicuous feature of the chromate-perturbed M9L proteome profile was the differential expression of proteins with functions in DNA replication, recombination and repair. An ATPase involved in DNA repair-like protein (Pput 2963), ATP-dependent DNA helicase Rep (Pput 5174), DNA repair protein RecN (Pput 4595), ribonuclease H (Pput 4172), and uracil-DNA glycosylase (Pput 4308) showed a high Poisson coefficient regression in response to chromate (Table 2 and Additional File 5). Peptides corresponding to the SOS-response transcriptional repressor LexA (Pput 3599) were detected only in control (untreated) M9L cell samples (Table 2), suggesting that the SOS pathway of DNA repair (which protects against oxidative stress) is activated in response to chromate exposure. Along with expression of the co-chaperone Hsp20, the up-regulation of DNA recombination and repair proteins indicates the greater toxic effect of chromate imposed on M9L-grown cells.

The two most highly represented COG categories (other than unknown function) for proteins with decreased abundance were associated with amino acid transport and metabolism (14 proteins) and energy production and conversion (17 proteins). A number of the down-regulated energy metabolism proteins had annotated functions associated with electron transport chain processes and included a proton-translocating NADH-quinone oxidoreductase (Pput 1736) and a cytochrome c (Pput 1947). It is also interesting to note that a NADPH-dependent FMN reductase (Pput 1727), which exhibits 100% amino acid sequence identity to the chromate reductase ChrR from *P*. *putida *strain KT2440, was detected at negligible levels in untreated (control) cell samples but was down-regulated in response to acute chromate treatment under M9L and LB growth conditions (Additional file 1). ChrR is a soluble dimeric flavin mononucleotide-binding flavoprotein that was shown to catalyze full reduction of Cr(VI) to Cr(III) [19] and more recently, was demonstrated to exhibit quinone reductase activity, of importance in protecting *P*. *putida *cells against H_2_O_2 _stress [20].

### Growth media-independent proteomic responses to chromate

We compared the Cr(VI)-perturbed proteome profiles for LB- and M9L-grown *P*. *putida *F1 cells to identify shared molecular signatures (biomarkers) indicative of chromate stress. Comparative analysis indicated that eleven proteins detected only in Cr(VI)-treated cells were common for the two different growth conditions (Additional file 6). Of these eleven chromate-responsive proteins, seven were determined to be statistically significant based on an FDR cutoff of 0.05 and consisted of a two-component transcriptional regulator of the winged helix family (Pput 0287), fumarate lyase (Pput 0984), superoxide dismutase (Pput 0985), a TonB-dependent hemoglobin/transferrin/lactoferrin family receptor (Pput 1043), 4-hydroxythreonine-4-phosphate dehydrogenase (Pput 0436), a FAD-dependent oxidoreductase (Pput 5183) and ribosomal protein L36 (Pput 0508) (Table 3). The functional diversity of these seven proteins, which represent six different COG categories, indicates the complexity of the core cellular response to chromate insult, a response that is likely complicated by secondary or indirect effects. In addition, the very limited subset of shared proteins suggests that growth conditions substantially influence the global molecular response to chromate.

**Table 3:** Statistically significant proteins detected only under Cr(VI) conditions in LB- and M9L-grown F1

			Spectral Counts^a^			
			
Gene ID	Protein Function	Functional category	Control LB	Control M9L	Cr(VI) LB	Cr(VI) M9L
Pput 0287	two component transcriptional regulator	Transcription	0	0	8	4.5
Pput 0436	4-hydroxythreonine-4-phosphate dehydrogenase	Coenzyme transport and metabolism	0	0	4.5	7
Pput 0508	ribosomal protein L36	Translation of ribosomal structure and biogenesis	0	0	4.5	7.5
Pput 0984	fumarate lyase	Energy production and conversion	0	0	64.5	7.5
Pput 0985	Superoxide dismutase	Inorganic ion transport and metabolism	0	0	13	4
Pput 1043	TonB-dependent hemoglobin/transferrin/lactoferrin family receptor	Inorganic ion transport and metabolism	0	0	15.5	3.5
Pput 5183	FAD dependent oxidoreductase	Amino acid transport and metabolism	0	0	6.5	3

^a^Spectral counts refer to the average spectral counts from two independent MS runs with an FDR cutoff of 0.05.

The table lists proteins common to chromate stressed, M9L and LB grown cells. The proteins were selected on the basis of their Poisson regression score and an FDR of 0.05. Spectral counts for each protein show the proteins to be uniquely expressed in chromate-stressed cells only.

## Discussion

This global proteomic study was undertaken to interrogate growth media-based differences in the *P*. *putida *F1 molecular response to the stress of the oxyanion chromate, a widespread anthropogenic contaminant and a current focus of bioremediation efforts by the DOE. The ultimate aim was to identify proteome changes reflective of, although not necessarily specific to, early chromate stress in microbial cells irrespective of the nutritional environment. Our physiological investigations indicated that the impact of chromate toxicity was largely dependent on the growth medium. Rich (LB) medium, for example, was able to support F1 growth at input chromate concentrations at least 40-fold greater than any of the defined minimal M9-based media. Presently, it is not known whether this observation was due to differences in growth rate as a function of the medium [the initial growth rate of F1 was 3 to 5 times faster in LB than in M9 media (data not shown)], a higher bioavailability of Cr(VI) in minimal media, or a lack of sequestration of toxic reduced chromium forms by organic molecules in the media. Comparative analysis of the proteomic profiles generated in this study revealed that acute chromate exposure affects, either directly or indirectly, a wide range of cellular processes and functions, with some of the most profound changes in expression occurring among proteins with annotated functions in inorganic ion transport, amino acid transport and metabolism, cell wall membrane and envelope biogenesis, and energy production.

Certain hallmark features of the bacterial molecular response to chromate challenge are emerging with the increasing availability of transcriptomic and proteomic descriptions of Cr(VI)-challenged microorganisms. Comparative analysis of the LB- and M9L-derived proteomes described here revealed that an adaptive strategy employed by *P*. *putida *F1 in response to acute chromate exposure was up-regulation of proteins involved in sulfate transport (sulfate ABC transporter Pput 1565), cysteine biosynthesis (Pput 4421 and 4422 encoding sulfate adenylyltransferase large and small subunits, respectively), and the uptake and utilization of alternative sulfur sources such as aliphatic sulfonates (taurine dioxygenase Pput 0190 and ABC-type nitrate/sulfonate/bicarbonate transport systems protein Pput 0191) (Additional file 1 and Table 2). Because of its structural similarity to SO_4_^2-^, exogenous chromate (CrO_4_^2-^) competes with sulfate for transport across bacterial cell membranes via sulfate anion transport systems [21-24], leading to low internal levels of sulfur. Up-regulation of genes/proteins with annotated functions in sulfur transport and metabolism has been observed in other chromate-stressed *Gammaproteobacteria *[25-27] as well as in the highly chromate-resistant *Arthrobacter *sp. strain FB24, a high G + C actinobacterium [28]. Using two-dimensional gel electrophoretic analysis, Ackerley *et al. *[25] showed that chromate-challenged nonadapted *E*. *coli *K-12 cells contained increased abundance levels of CysK and CysN, while challenged pre-adapted cells expressed alkane sulfonate monooxygenase, which functions by converting alkane sulfonates to sulfite and aldehyde. Similarly, multidimensional HPLC-MS/MS analysis of *Shewanella oneidensis *MR-1 exposed to different sub-lethal concentrations of chromate demonstrated increased abundance of cysteine synthase A (CysK), sulfate adenylyltransferase (CysD and CysN), adenylylsulfate kinase (CysC), sulfite reductase (CysI), and periplasmic sulfate-binding protein (Sbp) [26,27]. A recent study investigating chromate resistance in *Pseudomonas corrugata *28, a Cr(VI)-hyper-resistant (MIC, 40 mM K_2_CrO_4_) bacterium, demonstrated that Cr(VI) susceptibility was attributed to insertional inactivation of *oscA*, which encodes a hypothetical small protein of unknown function and is located in a gene cluster with components of the sulfate ABC transporter system [29]. We previously reported that *S*. *oneidensis *SO4651, a homolog of OscA [29], was up-regulated at both the transcript and protein levels during the entry response to Cr(VI) stress [26]. Viti *et al. *[29] showed that the *P*. *corrugata oscA*-*sbp *transcriptional unit was strongly overexpressed after chromate exposure, thus lending further support for the link between sulfate starvation and Cr(VI) stress response. Sufficient cysteine availability is critical not only for protein biosynthesis but also in the defense against metal-induced oxidative stress by maintaining cellular redox homeostasis through the production of protective thiol-containing compounds such as glutathione. Therefore, the up-regulation of genes/proteins involved in sulfur transport and metabolism constitutes a major and conserved cellular response among *Gammaproteobacteria *to the oxidative burden imposed by chromate challenge.

Reduced *P*. *putida *F1 growth rates and higher sensitivity to chromate in M9L medium may be attributable to stress resulting from low intracellular sulfate levels as well as high intracellular chromate levels. The sole source of sulfate in M9L medium is MgSO_4_, which is likely transported into the cell by the sulfate active transport system. This transport system has been shown in other *Pseudomonas *species to be principally responsible for the uptake of chromate [24]. As already mentioned, chromate is a competitive inhibitor of sulfate uptake [24]. Reduced sulfate uptake is compensated for by increased uptake of cysteine and cysteine-containing compounds (as well as other sulfur-containing compounds) through alternative transporter systems not competitively inhibited by chromate. Cysteine is freely available in complex LB medium but not in M9L medium. The reduced availability of cysteine in M9L medium could have resulted in lower levels of intracellular cysteine needed for important cellular biosynthetic processes, leading to the more striking reduced growth rates and chromate sensitivity of cells cultivated in M9L medium compared to LB medium.

Chromate toxicity has been attributed primarily to oxidative stress generated by the intracellular reduction of Cr(VI) to the transient highly reactive radical Cr(V), which redox cycles and thereby creates reactive oxygen species (ROS) [22,25,30]. ROS production leads to the damage of such cellular components as DNA and proteins. Presumably to counter Cr(VI)-induced oxidative stress, synthesis of the antioxidant defense protein Mn/Fe-binding superoxide dismutase (Sod; Pput 0985), which catalyzes decomposition of superoxide anion (O_2_·^-^) to hydrogen peroxide and molecular oxygen, was detected only in Cr(VI)-exposed LB- and M9L-grown *P*. *putida *F1 cells. Similarly, the up-regulation of Sod at the transcript or protein level was observed for Cr(VI)-stressed *E*. *coli *[25] and the *Alphaproteobacterium Caulobacter crescentus *[31] but not for *S*. *oneidensis *MR-1 cells subjected to an acute chromate treatment [26]. Instead, *katG-1 *(catalase/peroxidase hydroperoxidase) and *katB *(catalase) were preferentially induced at Cr(VI) exposure time intervals of 60 and 90 min. Based on our proteomic analyses, the enhancement of free-radical detoxifying activities constitutes a vital cellular defense mechanism against chromate toxicity in *P*. *putida *F1 regardless of the growth medium, whereas cellular defenses against H_2_O_2 _(*i.e.*, increased expression of catalase) were not required during the early stages of chromate exposure in contrast to that observed for *S*. *oneidensis *MR-1.

Located immediately upstream of the F1 *sod *gene is *fumC *(Pput 0984), which also was identified only in Cr(VI)-stressed cells under both growth media conditions (Table 3). The gene synteny is similar (but not identical) to that found in the genome of *Pseudomonas aeruginosa*, in which an O_2_·^- ^resistant isoform of fumarase (or fumarate hydratase/lyase) is linked to downstream genes *orfX *(of unknown function) and *sodA *(encodes Mn-cofactored Sod) in an iron-responsive operon [32]. Fumarase catalyzes the reversible conversion of fumarate to malate in the TCA cycle and has been shown to be up-regulated under iron-limiting conditions in *P*. *aeruginosa *[33]. It remains to be determined whether expression of *P*. *putida *F1 FumC exclusively in Cr(VI)-challenged cells is part of an iron starvation response.

As previously demonstrated for *S*. *oneidensis *MR-1 [26,27] and *C*. *crescentus *[31], another prominent characteristic of the cellular response to acute chromate exposure is the up-regulation of genes/proteins with functions in iron acquisition and homeostasis, namely TonB-dependent receptors and siderophore biosynthesis proteins. Similarly, TonB-dependent receptors for high-affinity iron chelators were a conspicuous feature of up-regulated, Cr(VI)-perturbed *P*. *putida *sub-proteomes, particularly under LB growth conditions. The increased abundance of four such outer membrane receptors under LB-Cr(VI) conditions is likely connected with the concomitant up-regulation of five amino acid adenylation domains. The adenylation (A) domains represent the active core of each modular unit comprising large multifunctional enzymes termed nonribosomal peptide synthetases [34]. The A domain functions by recognizing a specific amino or hydroxyl acid substrate and activating it as aminoacyl adenylate via ATP hydrolysis. While nonribosomal peptide synthetases can produce peptides of broad and sometimes obscure biological activity, these multidomain enzymes are known to be involved in the assembly of aryl-capped peptide and peptide-polyketide siderophores from *Pseudomonas *spp. [35,36]. Furthermore, expression of a TonB-dependent hemoglobin/transferrin/lactoferrin family receptor (Pput 1043) as part of the media-independent core molecular response to chromate exposure points to iron availability and homeostasis as playing an important role in the cellular adaptation of F1 to chromate stress.

Earlier reports revealed a distinct link between cell sensitivity to chromate and iron availability. It was observed, for example, that *tonB *mutants of *E*. *coli *exhibited increased sensitivity to chromium salts [37-39], which could be relieved by adding iron to the growth medium [38]. More recently, siderophores produced by certain *Pseudomonas *species were shown to bind exogenous transition metals other than Fe(III) with appreciable affinity [40,41], thus invoking conditions of low internal iron. The siderophore pyridine-2,6-bis(thiocarboxylic acid) (pdtc) from *Pseudomonas stutzeri *KC, for example, has been shown to detoxify extracellular chromium(VI), selenium, and tellurium oxyanions, suggesting that pdtc functions not only in iron acquisition but also in an initial line of defense against metal toxicity [42,43]. Research is required to delineate the physiological basis for the increased expression of iron acquisition receptors in response to acute chromate stress.

The Cr(VI)-responsive proteomic subset shared between the two different growth conditions consisted mostly of structural proteins with the exception of one regulatory protein, annotated as a two-component response regulator (Pput 0287) belonging to the winged helix-turn-helix family of DNA-binding regulators. Prototypical two-component systems, which constitute the predominant mechanism used by bacteria for coupling environmental signals to specific adaptive responses, comprise a sensor histidine kinase and a cognate response regulator [44,45]. The regulatory targets for the *P*. *putida *response regulator have not previously been identified. A similar observation was noted for Cr(VI)-stressed *S*. *oneidensis *MR-1 cells in which a DNA-binding response regulator (designated SO2426), also of the OmpR/PhoB subfamily [46] of winged-helix DNA-binding domains, was detected at the protein level only in chromate-challenged cells [26,27]. Further in-depth functional analysis using a Δso2426 mutant strain found that this two-component response regulator likely is involved in the activation of genes required for siderophore-mediated iron acquisition [47]. A DNA-binding response regulator consisting of a CheY-like receiver domain also was up-regulated (at the transcript level) in Cr(VI)-treated *C*. *crescentus *[31]. At this point, it is not known whether expression of Pput 0287 is part of a cellular regulatory system for sensing external toxic metals or a secondary effect of Cr(VI) challenge.

## Conclusions

In summary, effective bioremediation strategies should encompass an understanding of the fundamental physiological and molecular processes extant in the remediating populations. Ideally, one would like to perform such studies in the most ecologically relevant context, but the trade-off is that *in situ *it is difficult to control enough variables to make the results interpretable. As described here, we have monitored growth and proteome expression in the laboratory under disparate but controlled nutrient conditions ranging from the less (a rich medium) to the more ecologically relevant (minimal medium). We have shown that a strain of *P*. *putida*, F1, well-known for its ability to degrade aromatic xenobiotics, is also resistant to low mM levels of Cr(VI) and is capable of reducing hexavalent chromium. Furthermore, the proteome expressed in response to acute chromate challenge is dependent upon factors other than just the presence of external chromate. Most importantly, we have identified in this study a core set of proteins that are differentially expressed in response to Cr(VI) challenge that appear to be media-independent, and this core protein subset will serve as a baseline for comparison with future proteomic analyses of *P*. *putida*-containing microbial communities in naturally occurring Cr(VI)-contaminated soils.

## Methods

### Strains, media, and growth conditions

*Pseudomonas putida *strain F1 (ATCC 700007) was grown aerobically at 30°C with vigorous shaking (200 rpm) in either Luria-Bertani (LB) medium or M9 minimal medium [2 mM MgSO_4_, 0.1 mM CaCl_2_, and 1X M9 salts (5X M9 salts contains per liter: 15 g KH_2_PO_4_, 2.5 g NaCl, 5 g NH_4_Cl, 64 g Na_2_HPO_4_7H_2_O)] supplemented with 50 mM (final concentration) of glucose, sodium lactate, or sodium acetate. Cultures of *P. putida *F1 were initially propagated in LB broth and then were passed twice (preconditioned) through M9 media amended with the appropriate carbon source prior to experiments using those media. The water used in these experiments was purified using a NANOpure Diamond system (Barnstead International, Dubuque, IA) and sterilized by autoclaving or passage through a 0.2 μm filter (VWR, West Chester, PA).

To determine the minimal inhibitory concentration (MIC) of Cr(VI) for *P. putida *F1 growth in rich and minimal media, five ml of LB broth or M9 medium plus a single carbon source (50 mM glucose, sodium lactate, or sodium acetate) was inoculated with a freezer stock of preconditioned *P. putida *F1 and grown overnight at 30°C with constant shaking at 200 rpm. Cultures containing growth medium and increasing concentrations of K_2_CrO_4 _were inoculated to obtain an initial optical density at 600 nm (OD_600_) of ~0.04 using 16-h overnight cultures as inocula. End-point turbidity measurements were recorded after 48 h of growth, and all MIC determinations were performed using three biological replicates.

For comparative proteomic analysis of chromate-treated cells in complex versus defined minimal media, five ml of LB or M9L (M9 medium plus 50 mM sodium lactate) were inoculated with a freezer stock of preconditioned *P. putida *F1 and grown overnight. These cultures were used to inoculate 300 ml of the same medium in 1 L flasks to an optical density of ~0.008 OD_600 _units. Cells were incubated at 30°C with constant shaking (200 rpm) until cultures reached mid-log phase (OD_600_~0.4 for M9L and OD_600_~0.6 for LB). Cultures were divided into two equal fractions: K_2_CrO_4 _was added to one fraction to a final concentration of 1 mM, while the other fraction served as the untreated (no chromate) control. Incubations were then continued under the same conditions for an additional 75 min after which time the cells were harvested by centrifugation at 3,000 × *g *for 10 min at 4°C. The supernatant was decanted and the cell pellet was resuspended in 100 ml of sterile 1× PBS buffer. The centrifugation and suspension were repeated as described prior to storage at -80°C until needed for proteome analysis.

### Chromate reduction assay

Chromate reduction levels were measured as described by Shmieman *et al. *[48]. Standards were prepared by the addition of K_2_CrO_4 _to sterile growth medium (LB medium, or M9 amended with one of the carbon sources described above) to final concentrations ranging from 0.1 to 1.0 mM (in 0.1 mM increments). A ChromaVer3 pillow was added to 9 ml of water and dissolved by vortexing, and 0.9 ml of the resulting ChromaVer3 solution was added to 0.1 ml of standard or *P. putida *F1-containing sample and mixed. The reactions were incubated for 15 min at room temperature after which time the absorbance at 540 nm was read in a Beckman DU 530 UV/Vis Spectrophotometer (Beckman Coulter Inc., Fullerton, CA) and the amount of Cr(VI) in each experimental sample was calculated from the standard curve. For each medium, a cell-free sample was monitored in parallel with the experimental cultures to control for abiotic Cr(VI) reduction.

### Sample preparation

*P*. *putida *cultures harvested for shotgun proteomic analysis were lysed by sonication in 50 mM Tris/10 mM EDTA (pH 7.6) as described previously [26]. Cellular debris was removed by centrifugation (5,000 × *g *for 10 min), and the soluble proteome fraction was separated from the membrane fraction by ultracentrifugation (100,000 × *g *for 60 min). The pellet (membrane fraction) was washed with 50 mM Tris/10 mM EDTA (pH 7.6) and centrifuged at 100,000 × *g *for 60 min; this fraction was then resuspended in 50 mM Tris/10 mM EDTA (pH 7.6) using brief sonication. Both proteome fractions (soluble and membrane associated) were quantified using the bicinchoninic acid (BCA) assay (Pierce, Rockford, IL), aliquoted, and maintained at -80°C prior to protein digestion. Equal protein amounts (1 mg) of each proteome fraction from each growth condition were diluted in 6 M guanidine and 10 mM dithiothreitol (DTT) followed by heat treatment (60°C, 1 h). The proteome fractions were then digested with a 1:100 (wt/wt) amount of sequencing grade trypsin as described previously [26]. Following proteolytic digestion, samples were desalted using C_18 _Sep-Pak solid phase extraction cartridges (Waters, Milford, MA), concentrated to ~10 μg/μL starting material, and solvent exchanged with 0.1% formic acid in HPLC grade water using a Savant SpeedVac (ThermoFisher Scientific, Waltham, MA). Samples were filtered, aliquoted, and stored at -80°C until ready for two-dimensional (2-D) LC-MS/MS analysis.

### 2D-LC-MS/MS measurements

The digested proteome fractions (soluble and membrane) prepared from *P*. *putida *cells (cultivated in LB or M9L minimal medium, with or without 1 mM chromate) were analyzed in duplicate via 2-D LC-MS/MS analysis using an Ultimate HPLC system (LC Packings, a division of Dionex, San Francisco, CA) coupled to a linear trapping quadrupole (LTQ) mass spectrometer (ThermoFisher Scientific, San Jose, CA). Details of the MS instrumentation are described elsewhere [26]. All samples were analyzed via a 24-h 12-step 2-D analysis consisting of increasing concentration (0-500 mM) salt pulses of ammonium acetate followed by 2-h reverse phase gradients from 100% aqueous solvent [95% H_2_O/5% acetonitrile (ACN)/0.1% formic acid] to 50% organic solvent (30% H_2_O/70% ACN/0.1% formic acid). During the entire chromatographic process, the LTQ was operated in a data-dependent MS/MS mode as described in detail in Brown *et al. *[26].

### Proteome bioinformatics

A protein database was created by combining the most recent version of the *P*. *putida *F1 genome database (available at the JGI website http://img.jgi.doe.gov/cgi-bin/pub/main.cgi) containing a total of 5252 predicted proteins with 36 common contaminants (trypsin, keratin, etc.). For all database searches, the MS/MS spectra were searched using SEQUEST [49] with the following parameters: enzyme type, trypsin; parent mass tolerance, 3.0; fragment ion tolerance, 0.5; up to four missed cleavages allowed; fully tryptic peptides only as described previously [26]. The output data files were then filtered and sorted using the DTASelect algorithm [50] using the following parameters: fully tryptic peptides only with ΔCN of at least 0.08 and cross-correlation scores (Xcorr) of at least 1.8 (+1), 2.5 (+2), and 3.5 (+3). These threshold scores have been tested rigorously in our laboratory and provide a high confidence of identification with a maximum false-positive rate of 1-2% [51]. Post-translational modifications and other fixed modifications were not included in the search parameters. The predicted proteome database, all resulting DTASelect files and extract files, as well as links to all identified spectra, are available at this website: http://compbio.ornl.gov/pseudomonas_putida_F1/chromate_response. The DTASelect results derived from LB and M9L media-grown cells were compared using the Contrast program [50] for each control and treatment condition. These results are located under the global contrast heading on the analysis page, as well as extract files with spectra, peptides and % coverage for each protein identification.

### Statistical analysis

The resultant proteome datasets were analyzed using the Poisson regression model as previously described [51]. The Poisson regression model is commonly used for count data, assuming that the data have a Poisson distribution and are relevant for differential proteome dataset analysis. In this study, the spectral counts for a protein in an experiment constituted the count data. As we evaluated medium and treatment effects separately, we only had a single independent variable in each model (medium or treatment). To make the spectral counts comparable across different experiments, the spectral counts for a protein were normalized to the total spectral counts for that given dataset. In Poisson regression, this is handled by adding the logarithm of total spectral counts as an independent variable with a fixed coefficient of 1. The *p *values generated by the model were further adjusted using the Benjamini and Hochberg correction to account for multiple comparisons [52]. An adjusted *p*-value of 0.01 (i.e., 1% False Discovery Rate, FDR) combined with a 2-fold change in expression were used to select for proteins that were differentially expressed between the two groups under comparison.

## Authors' contributions

DKT and GSW primarily conceived the study, helped prepare cell samples for proteomic analyses, supervised all physiological experiments, and performed the majority of the data interpretation and manuscript writing. KC performed the proteomic measurements, participated in the data interpretation, and helped to develop the proteomic datasets along with MS and NCV. SBT, ATM, and MAR carried out all of the physiological studies and chromate reduction assays for *P*. *putida *F1. BZ conducted the statistical analyses of the proteomic data and provided text for this portion of the manuscript. NCV and RLH coordinated and supervised the mass spectrometry studies. All authors read and approved the final manuscript.

## Supplementary Material

Additional file 1 **Complete proteome data and dataset comparisons with statistical analysis**. Complete dataset detailing the spectral counts for each treatment and its technical replicate. The extended table shows statistically analyzed comparisons between two treatments. Proteins that are highlighted show a two-fold change in expression and 1% False Discovery Rate (FDR). Click here for file

Additional file 2 **Proteins detected in all technical replicates for cells grown in LB or M9L media**. The table shows proteins detected in all technical replicates of cells grown in LB or M9L media [control and Cr(VI) shock]. Click here for file

Additional file 3 **Proteins unique to LB control compared to M9L control**. Table lists proteins unique to unstressed LB grown cells compared to cells grown in M9L medium. Click here for file

Additional file 4 **Comparison of proteins expressed in unstressed cells grown in LB versus cells grown in M9L medium**. Comparison of total proteins expressed in unstressed LB grown cells versus cells grown in M9L medium. Click here for file

Additional file 5 **Differential protein profile in control and Cr(VI)-shocked M9L-grown cells**. Datasheet shows differential protein profile of control and Cr(VI)-shocked cells grown in M9L medium. Click here for file

Additional file 6 **Unique proteins induced in LB-grown cells and M9L-grown cells in response to chromate**. Table lists proteins uniquely expressed under Cr(VI) stress in LB and M9L grown cells. Click here for file
